# Farm typology of smallholders integrated farming systems in Southern Coastal Plains of Kerala, India

**DOI:** 10.1038/s41598-021-04148-0

**Published:** 2022-01-10

**Authors:** Anitrosa Innazent, D. Jacob, J. S. Bindhu, Brigit Joseph, K. N. Anith, N. Ravisankar, A. K. Prusty, Venkatesh Paramesh, A. S. Panwar

**Affiliations:** 1 Department of Agronomy, College of Agriculture, Kerala Agricultural University, Vellayani, Thiruvananthapuram, Kerala 695 522 India; 2 On-Farm Research Centre, College of Agriculture, Kerala Agricultural University, Vellayani, Thiruvananthapuram, Kerala 695 522 India; 3 Department of Agricultural Statistics, College of Agriculture, Kerala Agricultural University, Vellayani, Thiruvananthapuram, Kerala 695 522 India; 4 Department of Agricultural Microbiology, College of Agriculture, Kerala Agricultural University, Vellayani, Thiruvananthapuram, Kerala 695 522 India; 5 ICAR-Indian Institute of Farming System Research, Modipuram, Meerut, 250 110 India; 6 ICAR-Central Coastal Agricultural Research Institute, Old Goa, Goa 403 402 India

**Keywords:** Ecology, Environmental sciences, Environmental social sciences

## Abstract

Adoption of an integrated farming system (IFS) is essential to achieve food and nutritional security in small and marginal holdings. Assessment of IFS to know the resource availability and socio-economic condition of the farm household, farm typology plays a critical role. In this regard, a sample survey of 200 marginal households practicing mixed crop-livestock agriculture was conducted during 2018–2019 at Southern Coastal Plains, which occupies 19,344 ha in Thiruvananthapuram district, Kerala, India. Farming system typology using multivariate statistical techniques of principal component analysis and cluster analysis characterized the diverse farm households coexisting within distinct homogenous farm types. Farming system typology identified four distinct farm types viz*.* resource constrained type-1 households with small land owned, high abundance of poultry, very low on-farm income, constituted 46.5%; resource endowed type-2 households oriented around fruit and vegetable, plantation crop, with a moderate abundance of large ruminant and poultry, high on-farm income, constituted 12.5%; resource endowed type-3 household oriented around food grain, extensive use of farm machinery, with a moderate abundance of large ruminant, low on-farm income, constituted 21.5%; and resource endowed type-4 household oriented around fodder, with high abundance of large ruminant, medium on-farm income, constituted 19.5% of sampled households. Constraint analysis using constraint severity index assessed the severity of constraints in food grain, horticulture, livestock, complementary and supplementary enterprises in each farm type, which allowed targeted farming systems interventions to be envisaged to overcome soil health problems, crops and animal production constraints. Farming system typology together with constraint analysis are therefore suggested as a practical framework capable of identifying type-specific farm households for targeted farming systems interventions.

## Introduction

Indian agriculture is facing multiple problems viz*.* soil health deterioration, stagnant productivity, a declining water table, soil salinity, decline in factor productivity, and a virtual halt in further expansion of the irrigated area^1–3^. The single enterprise-based research efforts made in the past were not sufficient to ensure future productivity gains in Indian agriculture. An integrated farming system (IFS) can bring about a substantial and sustained increase in agricultural production ensuring the livelihood of farm households^4–6^. The farming system is a population of individual farm households, its resources, resource flows, and interactions at individual farm level, that have broadly similar resource bases, enterprise patterns, household livelihoods, and constraints for which similar development strategies and interventions would be appropriate^7^. The farming system represents an appropriate combination of farm enterprises viz*.* cropping systems, horticulture, livestock, fishery, forestry, and poultry which adequately interact with the environment without dislodging the ecological and socio-economic balance while striving to achieve the national goals^8^. The farming system is well-positioned to play a key role in India successfully achieving United Nations’ Sustainable Development Goal-2 (SDG2), which seeks to end hunger, achieve food security, improve nutrition, and promote sustainable agriculture.

An IFS is a management strategy that greatly ensures optimal utilization of resources within the farming system to maximize productivity and profitability while ensuring sustainability. The major steps in the IFS approach are the characterization of the prevailing farming system, identification of production constraints, maximization of production and profits through cost-effective socially acceptable interventions to overcome the constraints^6^. Within the single farming system, there exists a considerable degree of heterogeneity that complicates the interpretation of constraints and detract from the overall development objective. The factors creating heterogeneity in the farming system are bio-physical viz, climate, soil fertility, slope, and socio-economic viz, preferences, prices, and production objectives^9^. Agricultural technologies with great potential will not be adopted by farm households if heterogeneity within the farming system is not addressed properly.

Typology which is the study of types, aims to identify farmers with common characteristics while accounting for farmer diversity and heterogeneity^10^. Farming system typology is a tool for in-depth farming system analyses and further exploratory studies for detailed characterization^11^. It helps to understand the factors that explain the adoption and rejection of new technologies^12^. It integrates quantitative, participatory, and statistical methods to summarize the large heterogeneous population of individual farm households by grouping them into a few coherent homogenous farm types^13^. The resulting farm types are conceptually meaningful, representative of the population, and easily identifiable within the population of individual farm households. Farm households coexisting within a farm type manage their farms similarly, have similar general strategies, face similar constraints, and have comparable opportunities. Numerous studies were carried out on characterization and constraint analysis of farming systems^14^. However, there is a paucity of information regarding the use of farming system typology for characterization and targeted farming systems interventions. With this background, the present study was undertaken with the objective to characterize farming systems with a focus on marginal farm households using typology, identify constraints as per-farm types, and envisage cost-effective socially acceptable farming systems interventions to overcome constraints.

## Materials and methods

The study was carried out during 2018–2019 at Southern Coastal Plains Agro-Ecological Unit (AEU), which occupies 19,344 ha (8.84%) of Thiruvananthapuram district, Kerala state, India ^15^. The geographical location of the study site is at 8°46’ N latitude, 76°53’ E longitude with altitude ranging from zero to 72 m above mean sea level. The climate of the region is tropical humid monsoon with an annual rainfall of 2360 mm, mean annual temperature of 27.6 °C, and soil moisture deficit period of nearly four months during summer. The major soil type of the region is coastal sandy soils, which are very deep, well-drained sands with very low cation exchange capacity, deficient in calcium, magnesium, and potassium^16^.

The methodological framework of farming system typology utilized in the study comprised of the following five steps. The first step was to formulate a hypothesis on the heterogeneity of farm households through focus group discussion with an expert ‘design panel’ of local stakeholders with good knowledge of the study area viz*.* Agricultural Officers in the Department of Agriculture Development and Farmers’ Welfare. Hypotheses formulated were stakeholder assumptions on main features of local agriculture, livelihood strategies, expected farm types, differences between farm types, and their relative proportions, which were used in the creation of a survey questionnaire to capture the heterogeneity of farm households. Generally, the major crops of the region are rice, vegetables, and plantation crops; the livestock component is dominated by dairy and poultry. The second step was to create baseline data of farm households through a sample survey. The survey questionnaire was used to interview 200 sample marginal farm households practicing mixed crop-livestock agriculture during 2018–2019 i.e. 20 households selected randomly from each of 10 panchayats selected purposively in the study area. The third step was to select from the surveyed data, key quantitative variables characterizing the farm households, through focus group discussion with the same local stakeholders who initially formulated hypotheses (Table 1).

**Table 1:** Key quantitative variables used to characterize and cluster the farm households into farm types.

Key quantitative variable	Farm type										*p*-value*
	1 (n = 93)		2 (n = 25)		3 (n = 43)		4 (n = 39)		Sample (N = 200)		
	Mean^α^	SEm ±	Mean^α^	SEm ±	Mean^α^	SEm ±	Mean^α^	SEm ±	Mean	SEm ±	
*Household*											
Members in household (Nos.)	4.01	0.068	4.44	0.232	4.02	0.113	4.00	0.116	4.12	0.058	0.218^NS^
Age of household head (years)	59.4	0.83	63.0	1.67	58.0	1.34	61.1	1.02	60.4	0.56	0.192^NS^
Land owned by household (ha)	0.34^b^	0.012	0.44^a^	0.034	0.47^a^	0.026	0.43^a^	0.022	0.42	0.011	0.007
*Labour*											
Household members working on-farm (Nos.)	1.02	0.015	0.96	0.040	1.00	0.033	1.03	0.026	1.00	0.013	0.403^NS^
Household members working non-farm (Nos.)	1.38	0.065	1.56	0.154	1.26	0.082	1.31	0.091	1.38	0.044	0.308^NS^
Use of farm machinery (h/year)^#^	0.09^c^	0.007	0.36^c^	0.053	4.43^a^	0.297	1.40^b^	0.141	1.57	0.071	0.009
*Land use*											
Foodgrain area (ha)^#^	0.01^c^	0.001	0.04^c^	0.006	0.41^a^	0.025	0.15^b^	0.016	0.15	0.007	0.002
Fruit and vegetable area (ha)^#^	0.15^b^	0.009	0.34^a^	0.025	0.03^c^	0.004	0.07^c^	0.008	0.15	0.007	0.004
Spice and condiment area (× 10^–1^ ha)	0.06	0.004	0.03	0.005	0.03	0.003	0.04	0.006	0.04	0.002	0.822^NS^
Plantation crop area (ha)	0.26^b^	0.011	0.34^a^	0.022	0.18^b^	0.014	0.26^b^	0.017	0.26	0.008	0.001
Fodder area (× 10^–1^ ha)^#^	0.01^c^	0.001	0.02^b^	0.003	0.04^b^	0.005	0.17^a^	0.014	0.06	0.003	0.006
*Livestock ownership*											
Large ruminant (LU)	0.09^c^	0.008	0.84^b^	0.119	0.87^b^	0.097	1.08^a^	0.078	0.72	0.035	0.003
Milch animal (LU)	0.05^c^	0.005	0.50^b^	0.078	0.53^b^	0.058	0.82^a^	0.057	0.48	0.024	0.004
Small ruminant (LU)	0.03	0.003	0.01	0.002	0.06	0.008	0.01	0.001	0.03	0.002	0.071^NS^
Poultry (LU)	0.25^a^	0.014	0.17^b^	0.022	0.05^c^	0.006	0.02^c^	0.003	0.12	0.006	0.004
*Livestock production*											
Milk (× 10^3^ L/year)	0.27^c^	0.026	3.12^b^	0.537	2.98^b^	0.368	3.84^a^	0.338	2.55	0.142	0.009
Egg (× 10^3^ Nos./year)	3.93^a^	0.216	2.99^b^	0.431	0.69^c^	0.086	0.55^c^	0.076	2.04	0.106	0.002
*Net income*											
Foodgrain income (× 10^3^ ₹)^#^	0.10^c^	0.010	0.62^c^	0.118	17.0^a^	1.92	4.75^b^	0.616	5.62	0.342	0.003
Fruit and vegetable income (× 10^3^ ₹)^#^	21.9^b^	1.61	58.9^a^	7.66	3.96^c^	0.513	9.75^c^	1.390	23.6	1.29	0.001
Spice and condiment income (× 10^3^ ₹)	0.47	0.044	0.29	0.046	0.10	0.012	0.13	0.019	0.25	0.015	0.916^NS^
Plantation crop income (× 10^3^ ₹)	13.4	0.89	19.2	2.57	15.5	1.63	14.3	1.51	15.6	0.73	0.218^NS^
Fodder income (× 10^3^ ₹)^#^	0.01^c^	0.001	0.08^b^	0.013	0.12^b^	0.014	0.57^a^	0.047	0.20	0.011	0.006
Large ruminant income (× 10^3^ ₹)	3.77^c^	0.367	38.0^b^	5.78	24.0^b^	2.74	54.0^a^	4.84	29.9	1.59	0.009
Small ruminant income (× 10^3^ ₹)	0.17	0.013	0.06	0.009	1.43	0.172	0.08	0.009	0.44	0.023	0.071^NS^
Poultry income (× 10^3^ ₹)	7.32^a^	0.471	3.40^b^	0.524	0.97^c^	0.135	0.39^c^	0.058	3.02	0.172	0.003
Crop income (× 10^3^ ₹)	35.9^b^	3.13	79.1^a^	12.34	36.7^b^	4.25	29.5^b^	3.45	45.3	2.49	0.006
Livestock income (× 10^3^ ₹)	11.3^c^	0.96	41.5^b^	6.72	26.4^b^	3.62	54.5^a^	6.11	33.4	1.91	0.007
Other farm enterprise income (× 10^3^ ₹)	0.14^b^	0.013	5.01^a^	0.631	0.15^b^	0.020	0.10^b^	0.013	1.35	0.076	0.005
On-farm income (× 10^3^ ₹)^#^	47.3^d^	4.27	125.6^a^	18.59	63.3^c^	8.21	84.1^b^	10.24	80.1	4.56	0.004
Off-farm and non-farm income (× 10^3^ ₹)	217	21.2	239	43.5	181	23.7	204	30.4	210	13.5	0.845^NS^
*Expense*											
All farm enterprises production cost (× 10^3^ ₹)^#^	69^b^	4.4	202^a^	29.1	179^a^	19.9	154^a^	17.5	151	7.4	0.003

^#^Variables included in principal component analysis; ^α^Bonferroni test, any two means having a common letter were non-significant; *Kruskal–Wallis test *p*-value < 0.05 were significant; ^NS^Non-significant; LU: livestock unit (cattle 0.5 LU, buffalo 0.5 LU, goat 0.1 LU, chicken 0.01 LU, duck 0.01 LU; Chilonda and Otte, 2006); *Foodgrain*: rice; *Fruit and vegetable*: banana, mango, amaranth, bitter gourd, brinjal, chilli, cowpea, okra, cassava, elephant foot yam; *Spice and condiment*: black pepper, ginger, turmeric; *Plantation crop*: coconut, rubber; *Fodder*: guinea grass, hybrid napier; *Large ruminant*: buffalo, cattle; *Small ruminant*: goat; *Poultry:* chicken, duck; *Milch animal*: lactating females of buffalo, cattle, goat; Other farm enterprise: complementary enterprise viz*.* apiculture, pisiculture and supplementary enterprise viz. nutritional kitchen garden, agro-processing and value addition.

**Figure 1:**
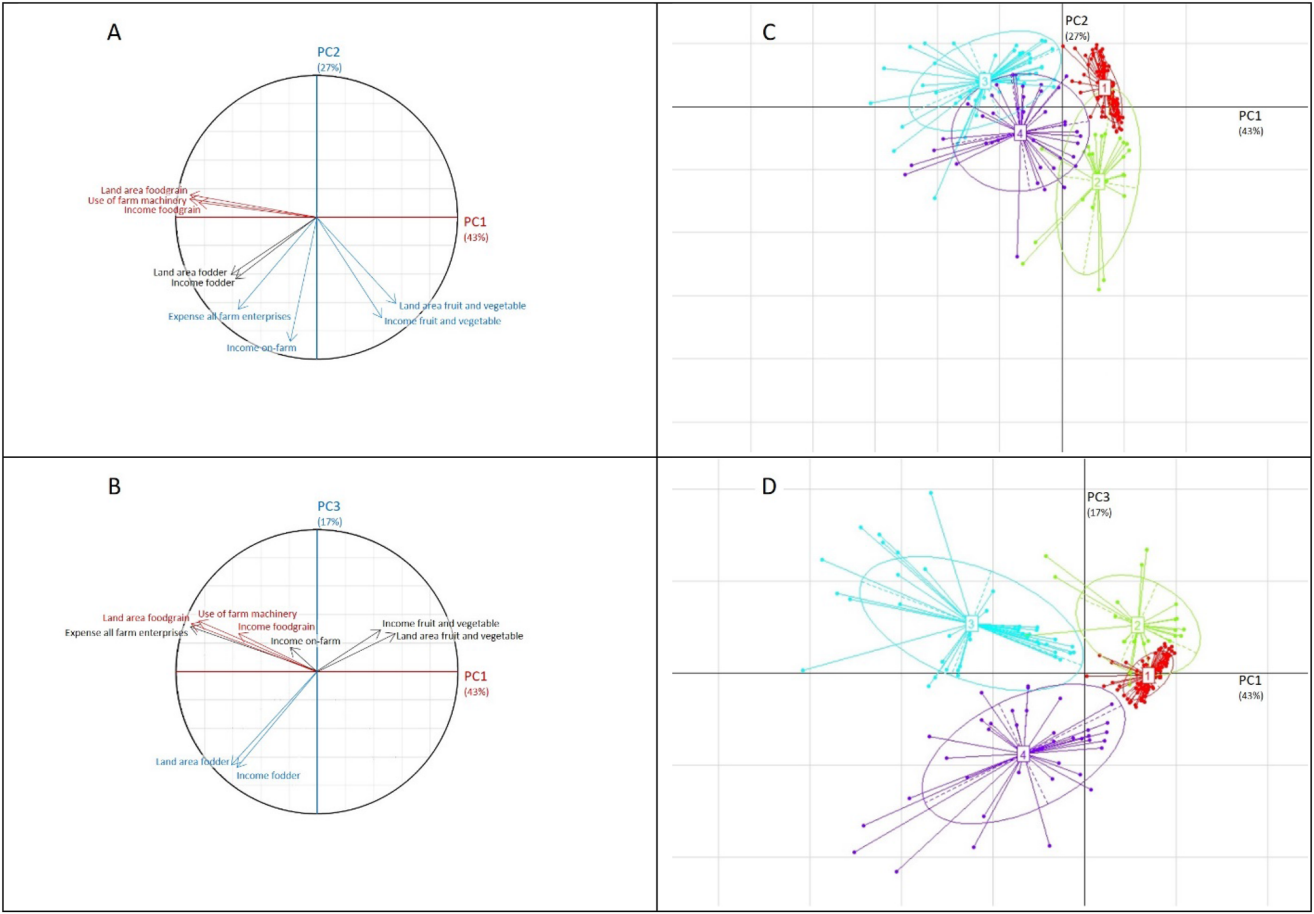
Spatial distribution of four farm types resulting from principle component analysis and cluster analysis on the planes defined by first three principle components: Circles of correlation (**A**, **B**) and clustered farm households viz*.*, farm types 1–4 (**C**, **D**) projected on the planes PC1–PC2 and PC1–PC3. The variables highlighted in red correlate strongly with PC1 and are the most explanatory variables of the horizontal axis (PC1); those variables highlighted in blue correlate strongly with PC2 and PC3 and are the most explanatory variables of vertical axes (PC2 and PC3), thus defining the gradients.

**Figure 2:**
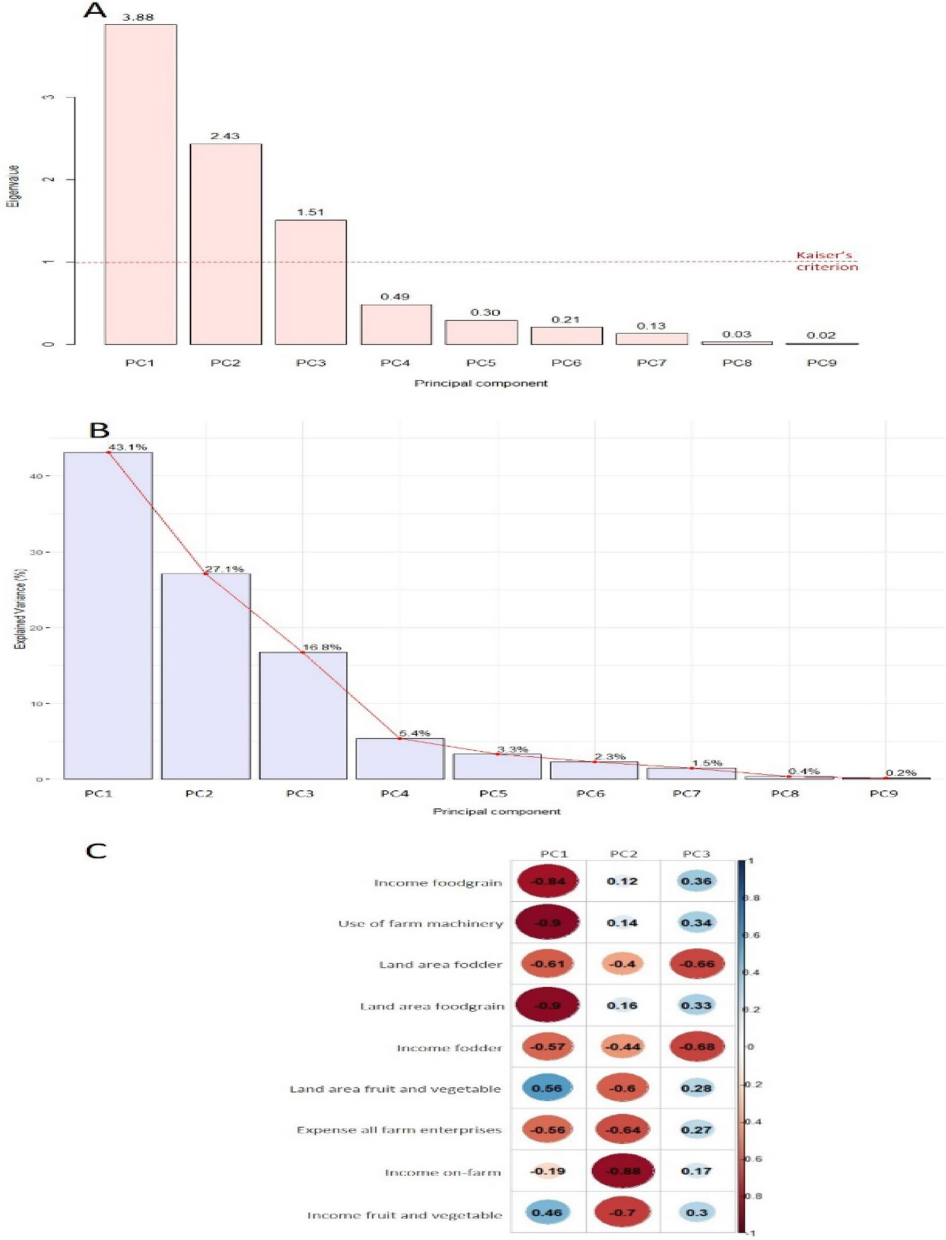
Principal component analysis: (**A**) Eigenvalue per principal component: Eigenvalues explained by successive principle components (PCs), the first three PCs that exceeded an eigenvalue of one represented by dashed line were retained based on Kaiser’s criterion; (**B**) Scree plot: Percentage variance explained by successive PCs, cumulative percentage of variance 87% explained by three retained PCs; (**C**) Correlation plot of PCs with variables.

**Figure 3:**
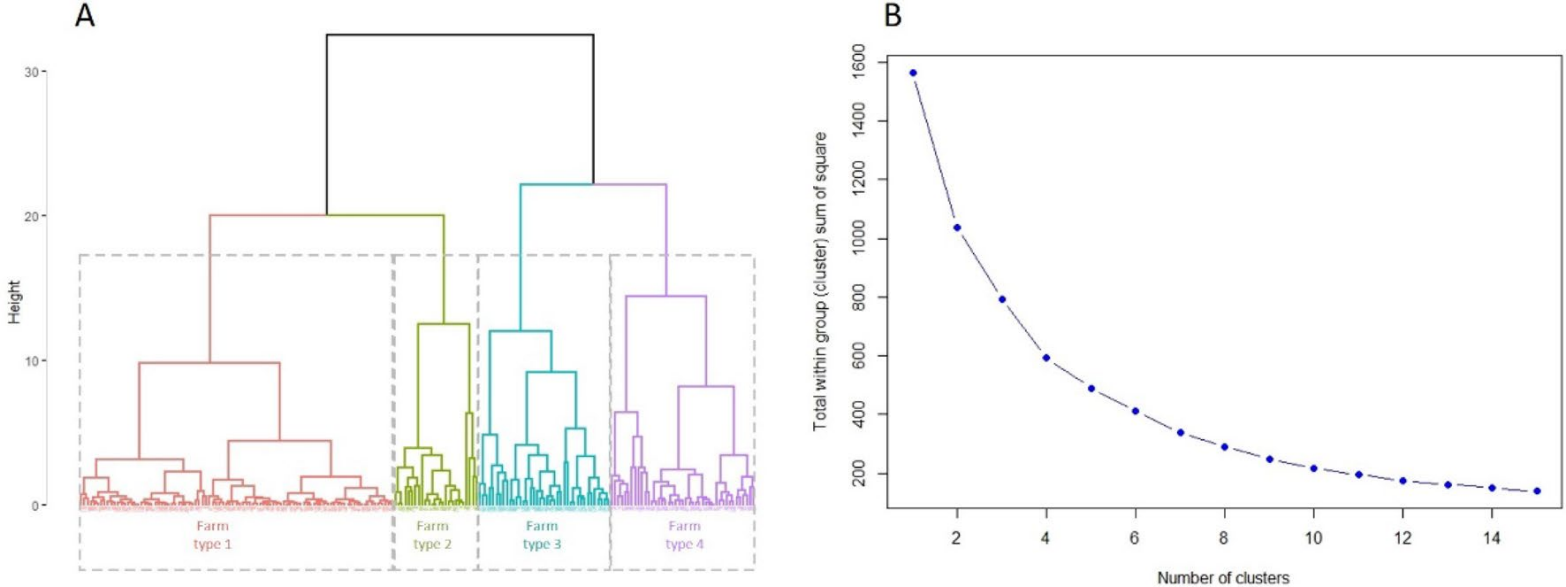
(**A**) Cluster dendrogram from agglomerative hierarchical clustering using the Ward’s method suggested four clusters; (**B**) Scree plot to determine optimal number of clusters also supported four clusters.

The fourth step was to distribute surveyed farm households among clusters by sequentially using two multivariate statistical techniques namely principal component analysis (PCA) and cluster analysis (CA) as shown in Fig. 1. PCA was used to reduce the key quantitative variables into a few principal components (PCs). The number of PCs to be retained was decided based on Kaiser’s criterion where all PCs exceeding an eigenvalue of one were initially retained (Fig. 2A). This decision was cross-checked by looking at the minimum cumulative percentage of variance chosen, here 87% (Fig. 2B). The interpretability of the conceptual meaning of PCs was assessed by examining the correlations between the variables and the PCs (Fig. 2C). PCA was followed by CA (Fig. 3). The PC scores from PCA were subjected to agglomerative hierarchical clustering using Ward’s minimum variance method to define the number of clusters and represented them by a dendrogram (Fig. 3A and B). A non-hierarchical clustering was subsequently performed to refine the number of clusters retained from Ward’s method and thus optimize the distribution of farm households among clusters. The clustered farm households were then projected on the gradient defined by PCs which allowed for distinguishing the farm types (Fig. 1C and Fig. 1D). The clustered farm households are referred to as farm types. Four farm types were identified in the present study (Table 1).

Farm type profiling of key quantitative variables using descriptive statistics was done to understand the most conspicuous characteristics of farm types and to compare the behavior of key quantitative variables between farm types (Table 1). Kruskal–Wallis test was used to assess significant differences in key quantitative variables (Table 1). Bonferroni posthoc test was used to identify which farm types were significantly different for each key quantitative variable (Table 1). The fifth step was to compare the farm types with an initial formulated hypothesis to confirm that farm types were conceptually meaningful having explanatory value, thus validating the hypothesis while ensuring wider acceptance and usability of the results. This validation was done through focus group discussion with an expert ‘validation panel’ of local stakeholders embedded in the population of individual farm households viz*.* farmers, who were potential users of the typology results. The farming system typology used in the study is in accordance with the methodology suggested by Alvarez et al*.*^17^, Kuivanen et al*.*^11^, and Alvarez et al*.*^13^. Experimental data recorded during the investigation were tabulated and analyzed in R 3.6.2 statistical software as elaborated by Barba-Escoto et al*.*^18^ to draw a valid conclusion.

Farm households in each farm type faced problems in agricultural production due to constraints that prevented the solution to the problem. Farmers declared 37 problems and constraints in the survey questionnaire and also assigned weightage for the severity of constraints as per farmer’s order of importance. After the focused group discussion and individual survey, the identified constraints validity was done with Agricultural Officers in the Department of Agriculture Development and Farmers’ Welfare, Kerala. Constraint Severity Index (CSI) was calculated using the formula,

$$\mathrm{CSI}=\frac{\sum_{i=1}^{n}\mathrm{w}i\mathrm{f}i}{\sum \mathrm{f}i},\mathrm{ i}=\mathrm{0,1},2, ...5$$

where, w*i*, weightage for the severity of constraints falling in the category of None, Very Low, Low, Medium, High, and Very High which were assigned a weightage of 0, 1, 2, 3, 4, and 5 respectively and f*i*, frequency of constraints. The rating was assigned to CSI. CSI zero was rated None, 0.1 to 1.0 Very Low, 1.1 to 2.0 Low, 2.1 to 3.0 Medium, 3.1 to 4.0 High, and 4.1 to 5.0 Very High. Higher the CSI, the more severe the constraint. CSI was developed from the Index of Constraints (CI), a formula described by Singh et al*.*^14^ for the identification of constraints, which assigned weightage on a predefined scale of one to ten. CSI differs from CI in the use of weightage from zero to five. No constraint has been largely excluded in CI, while it was accounted for in CSI. The rating assigned to CI also differs from CSI. Based on constraints to agricultural production identified, targeted farming systems interventions to achieve desired developmental objectives were envisaged as per the standard package of practices recommendations to overcome the constraints.

### Informed consent

Required informed consent were obtained from farmers during survey time, as it is a survey type of research.

## Results and discussion

### Characterization of farm types

The principal component analysis (PCA) resulted in extraction of the first three principal components (PCs) based on eigen-value criterion (eigen-value > 1) (Fig. 2A) explaining about 87% of the variability in surveyed farm households (Fig. 2B). The first principal component (PC 1) explained the greatest part of the variation, about 43.1% of the variability in surveyed farm households. PC 1 was more closely related to the variables describing the use of farm machinery, land area foodgrain, and income foodgrain. (Fig. 1A and Fig. 2C). The second principal component (PC 2) explained 27.1% of the variability in surveyed farm households and was strongly associated with land area fruit and vegetable, income fruit and vegetable, income on-farm, expense all farm enterprises (Fig. 1A and Fig. 2C). The third principal component (PC 3) explained 16.8% of the variability in surveyed farm households and described land area fodder, income fodder (Fig. 1B and Fig. 2C). Thus, the first three principal components explained the use of farm machinery, land use, income, and expense of farm households, giving insight into the production objective of households. The results from hierarchical clustering suggested a four-cluster cutoff point (Fig. 3A and Fig. 3B) and the non-hierarchical clustering assigned households to identified clusters (Fig. 1C and Fig. 1D). Thus households of the study area could be grouped into four farm types contrasted by their structural characteristics that describe resource endowment and functional characteristics that describe livelihood strategies. Traditionally, farm households were divided into four categories based on the size of their land holdings: marginal, small, medium, and large farmer^19^. The typologies created in this study are based on the possession of resources such as crops and animals, as well as decisions made by them regarding crop and livestock rearing. Based on structural factors, cropping system, livestock owned, source of income, and differences among different farm households, our study divided the farm households into four farm types. The similar type of categorization was done for smallholder’s farms in Indo‑Gangetic Plains of India^20^.

*Farm type-1. Resource constraint households with low farm income (n* = *93, 46.5%):* Farm type-1 was the largest cluster of sampled farm households, distinguishable from other farm types by smallest land owned by household (Table 1). The cropping system dominated by plantation crop, had fruits and vegetables. Nearly half of fruits and vegetables as sole crops and the rest are intercropped in coconut. The livestock system exhibited a low abundance of large ruminant and a high abundance of poultry, average ownership was limited to the isolated presence of cattle and 25 poultry. Egg production was highest among farm types. On-farm income were the lowest among farm types. Crop produce sales were the main source of on-farm income 76%, complemented by income from livestock 24%. Furthermore, the production cost of ₹69,000 was the lowest among farm types. Due to variables such as fluctuating commodity prices, labour shortages during peak agriculture season, farmers' concentration shifted to adoption of few enterprises as a result of land fragmentation and economic liberalization in the 1990s^21,22^. These variables have had a significant impact on resource constraint farm types.

*Farm type-2. Resource endowed diversified households with high farm income (n* = *25, 12.5%):* Farm type-2 exhibited the smallest cluster of sampled farm households, mostly dominated by fruit and vegetable, plantation crop (Table 1). Nearly one-fourth of fruit and vegetable as the sole crop and the rest are intercropped in coconut in upland. Complementary and supplementary enterprises viz*.* apiculture, pisciculture, nutritional kitchen garden, agro-processing, and value addition generated income ₹5,010 which was substantially high in this cluster. Livestock production centered around a moderate abundance of large ruminant and moderate abundance of poultry, average ownership of 1 cattle and 17 poultry. This cluster had the highest on-farm income ₹1,25,600 among farm types. Crop produce sales provided 63% of on-farm income, complemented by income from livestock 33%. Moreover, the production cost of ₹2,02,000 was relatively high among farm types. These farm households adapted crop diversification. Diversification is a method for making better use of land, water, and other resources by growing more profitable crops. It allows farmers to choose which crops to grow on their farm in order to maximize returns, and most farmers grow multiple crops to reduce risk and uncertainty caused by climatic and biological fluctuations^23^. Diversification refers to switching from less profitable and non-sustainable crops to more profitable and long-term crops. It has emerged as a viable option for ensuring natural resource sustainability, ecological balance, job creation, and risk generation^24^.

*Farm type-3. Resource endowed mechanized households with low farm income (n* = *43, 21.5%):* Farm type-3 comprised of sampled farm households distinguishable from other farm types by the largest cropped area under foodgrain (Table 1). The foodgrain area dedicated to rice cultivation was located mostly in wetland, while the plantation crop area largely established with coconut was on paddy field bunds and in the garden land. Livestock production centered around a moderate abundance of large ruminant and low abundance of poultry, average ownership of 1 cattle and 5 poultry. This cluster had an on-farm income of ₹63,300, the main source being crop produce sales 58%, complemented by income from livestock 42%. Besides, the production cost of ₹1,79,000 was relatively high among farm types. In these farm households the farm mechanization has brought significant change in the livelihood. Especially, paddy field preparation through puddling, mechanical transplantation, and paddy combine harvester reduced the greater dependence of external labourers. The relative shortage of agricultural workers, and the comparatively high wage rate in agriculture has bought small and large scale mechanization in Kerala agricultural system^21^.

*Farm type-4. Resource endowed medium farm income households with livestock dominance (n* = *39, 19.5%):* A main distinguishing feature of sampled farm households in farm type-4 was the largest fodder area among farm types, established mostly in coconut garden (Table 1). A considerable number of households had a foodgrain area of in wetland, mainly dedicated to rice cultivation. The livestock system exhibited a high abundance of large ruminant and low abundance of poultry, comprised mostly of milch animal, average ownership of 2 cattle and 2 poultry. Milk production 3.84 × 10^3^ L/year was the highest among farm types. On-farm income was ₹84,100. The main income source was livestock which constituted 65% of on-farm income, complemented by income from crop produces 35%. Production cost ₹1,54,000 was relatively high among farm types. These farmers adapted livestock has their source of livelihood and alternate means of employment especially farm women’s. The major benefit of livestock components like cattle and poultry is that they provide regular income to sustain farm family and also they provide nutritional security. Crossbred cattle adoption and crossbred milk output are important factors in increasing livestock revenue. To increase income from animal sources, a crossbreeding strategy should be implemented^25^.

### Farming system patterns

Distinguishing characteristics of a farming system are highly location-specific, depend on adaptive strategies devised by farmers to cope with the adverse situations as well as take advantage of the potential opportunities for intensification and diversification of agriculture at the household level. Studies have shown that farmers come up with strategies to get along with adverse situations viz*.* volatile price, crop failure, flood, drought, declining soil fertility, land scarcity, climate change and also make use of potential opportunities viz*.* use of new technologies, value addition, which allowed for sustainable production and income^10,26–28^. These distinguishing characteristics of a farming system are discussed in relation to clustering variables grouped according to the theme, their interrelationships, and the identified farm types in the following sections.

*Farm household:* The basic unit of social organization is the farm household where the head, typically a male lives with his nuclear family most often in a concrete roofed house. Farm households residing in traditional clay tile-roofed houses are also found occasionally. Farm households had an average size of four members (Table 1). Households were headed by the oldest male member aged 60 years. Both household size and age of household head remained unchanged across farm types. Land owned by households 0.42 ha is typically inherited (Table 1). Purchase is the less common access route to land ownership. Land owned by a household is commonly taken as a proxy for the wealth of a household as it correlates positively with livestock assets and crop production^29^. Results revealed variation in land owned by households across farm types with the smallest land 0.34 ha owned by the resource-constrained type-1 household. Interestingly, type-1 farmers accounted for a major proportion (46.5%) of farm households surveyed. The traditional practice of land owned by households typically fragmented into smaller parcels that are allocated to children at the time of their marriage, favors an increase in the number of small farm holdings. Eventually, the married children who had started in a household, leave the household with one’s spouse and consequently their children to build their own house and live separately in their inherited land, thus forming a new household. Small land holdings characterize Kerala agriculture. The core cause of poverty in Kerala is the tremendous fragmentation of agricultural land, and the fact that this fragmentation is only getting worse and is becoming a unique development issue. This current state of significant fragmentation, highlight the massive increase in the number of marginal farms as the area covered by large farms decreases^30^.

*Labour:* A combination of family and hired wage labour was used for agricultural production in the study area. Family labour is comprised of individuals in a household who are related by blood and kinship. With all households having only one family member working on-farm on a full-time basis and the average household size being only four members, family labour availability is less (Table 1). Household size is commonly taken as a proxy for family labour availability thereby requiring the hiring of wage labour to deal with family labour shortage ^11^. Shortage of family labour is further exacerbated by one member in each household across farm types working non-farm on a full-time basis, either making a livelihood from overseas, running small businesses, or earning a salary from the service sector. The study area is located on the outskirts of the state capital, the educated youth in farm households have ample employment opportunities in the secondary sector namely construction, and in the tertiary sector namely health service, transportation, education, entertainment, tourism, finance, sales, and retail. Wage labourers were hence hired on a seasonal basis for labour-intensive activities such as land preparation, planting, and harvesting. The local wage rate for farm laborers in the study area were ₹650 and ₹600 per man-day for men and women respectively, which were the highest in the nation. For farmers and labourers, agriculture is not a reliable source of revenue and employment. Kerala's labour distribution has shifted in favor of the non-agricultural sector, especially the service sector. Kerala has seen a significant increase in non-agricultural employment in both rural and urban areas, resulting in a shift in the workforce's industrial distribution. The structure of rural employment in Kerala has transitioned from agricultural to non-agricultural enterprises as a result of these changes. The specialized agriculture practices and mono-cropping increased production cost, risk of crop failure, and lower market price^31^. Due to this, the small and marginal farmers migrated to neighboring cities in search of jobs and livelihood. In this scenario, IFS will be a solution to reduce the economic risk with improved employment generation. The continuous labour requirement for multiple crops and livestock systems provides an option for higher employment generation and keeps the farm families engaged in the farm activities. This holds good even during the COVID-19 pandemic for meeting the employment needs of reverse migrants (urban to rural). In IFS, farm activities are continued round the year, thus the farm family is effectively engaged in farm. The adoption of such systems avoids migration of farmers and rural youth to nearby cities and towns in search of contractual employment.

Results showed increased use of farm machinery, 4.43 h/year in the type-3 household having a considerable land area under foodgrain (Table 1). Tractor-operated rotavator for puddling and combined harvester for reaping, threshing, and winnowing were extensively custom hired in the type-3 household. Mechanization in foodgrain cultivation was limited to custom hiring of tractor-operated rotavator for puddling in type-4 households resulting in the use of farm machinery1.40 h/year (Table 1). Brush cutter for trimming weeds, coconut tree climber for harvesting coconut, and plant protection sprayers were some of the machinery owned by a limited number of households across all farm types. The variables viz*.* use of farm machinery, land area under foodgrain, and net income from foodgrain sales were positively correlated, attributable to substitution of wage labourers with machines in agricultural enterprises having high work and maintenance requirements so that such enterprises remain economically viable (Fig. 1A, B; Table 1).

*Land use*: Coconut plantation in upland and rice in lowland is the major land use. The two crop variables retained for principal component analysis (PCA) namely foodgrain area, fruit, and vegetable area, were negatively correlated to each other, suggesting that farms that dedicated large areas to field crops especially rice cultivation did so at the expense of fruits and vegetable crops especially banana, amaranth, cowpea and vice versa (Fig. 1A and Fig. 1B ; Table 1). Resource-constrained type-1 and resource endowed type-2 households exhibited the smallest cropped area under foodgrain (Table 1). The meager food grain area in type-1 and 2 households were under direct-seeded upland rice, cultivated as part of the latest efforts to diversify the existing cropping system in these households. Rice is the most widely consumed staple in the study area. The lower proportion of food grain in these households suggests that land resources had been preferentially allocated for production-oriented towards high-value crops especially fruit and vegetables (Table 1). This may be partially explained by copious non-farm income generated by type-1 and 2 households and apparent re-investment of that income preferentially for high-value crops especially fruit and vegetables.

Results suggest that in resource-constrained type-1 and resource endowed type-2 households with ample off-farm and non-farm income having ensured access to market for foodgrain needs, land owned was preferentially allocated for production-oriented towards fruit and vegetables, to ensure nutritional security. It might have been otherwise utilized for land resource allocation in type-1 and 2 households had there been insufficient off-farm and non-farm income. A marginal shift from staple foodgrain to horticulture does not adversely affect food security at the household^32^.

Resource endowed type-3 and 4 households, though had sufficient off-farm and non-farm income comparable with type-1 and 2 households, did not follow this pattern, with foodgrain area being more abundant among them. This suggested that farm households that dedicated large areas to field crops especially rice cultivation did so due to land topography favoring the prolonged presence of water creating wetlands. The rice crop residues were utilized to reduce the feeding cost of high-valued large ruminants especially cattle maintained in type-3 and 4 households (Table 1). In addition to the utilization of rice crop residues as feed for large ruminants, type-4 households had a higher proportion of land area dedicated to fodder, reducing even further their feeding cost.

*Livestock:* The livestock species and their number owned represent the wealth of a farm household. Large ruminant cattle are the most valuable livestock. Small ruminant goats, though hardy and prolific, are less valued. Rearing of large and small ruminants is a crucial form of fortification against extreme shocks such as crop failure or medical emergency of household members, providing immediate cash. Results showed higher large ruminant ownership 1.08 LU in type-4 households (Table 1). Type-4 households recorded the highest milk production, followed by type-3 households, presumably due to higher fodder area in type-4 households leading to better feed quality and quantity, improved animal performance, and increased carrying capacity of cattle by maximizing stocking rate. The presence of state-owned milk marketing cooperative in the study area had played a role in the large ruminant ownership, due to the added advantage of assured steady market and stable milk price. Small ruminant ownership of 0.03 LU tended to be quite similar across farm types (Table 1).

Households in all farm types had poultry flock kept in the traditional backyard poultry system, as a source of quick cash and protein-rich food (Table 1). The traditional backyard poultry system is characterized by an indigenous night shelter system, a scavenging system with scant supplementary feed, natural hatching of chicks, low productivity of birds, local marketing, and minimal health care practices^24^. Results indicated that the size of the poultry flock tended to increase as farm resource endowment decreased (Table 1). Resource constrained type-1 household exemplified this, as it had the highest poultry flock size of 0.25 LU and exhibited the highest income from poultry sales. Poultry flock size tended to be quite low and similar in resource endowed type-3 and 4 households. Backyard poultry system due to its least demanding nature in terms of infrastructure has been widely accepted by resource constrained households, enabling them to make a profit from the sale of poultry products^11,33^. Relatively high income from poultry sales in type-1 and 2 households represent a coping strategy to prop up household finances to access the local market for foodgrain needs. Farm households depending on traditional backyard poultry generally lacked access to adequate low-cost organic fertilizers especially farmyard manure, resulting in low productivity of crops, which may further exacerbate food insecurity^28^.

*Income:* Shortfalls in agricultural production and thus agricultural income were common in the study area, compelling households to diversify their livelihoods. Sources of farm household income are on-farm, off-farm, and non-farm income^34^. On-farm income comprised of sales income from the crop, livestock, complementary, and supplementary enterprises (Table 1). Type-2 farm households recorded a high on-farm income of ₹1,25,600, as it befitted from a livelihood strategy of production of high valued fruit and vegetable in addition to plantation crops. Crop sales contributed 63% to on-farm income in type-2 farm households. Type-4 farm households recorded medium on-farm income ₹84,100, as it befitted from a livelihood strategy of production of fodder in addition to food grain and plantation crops. This resulted in increased carrying capacity and maximized stocking rate of large ruminant 1.08 LU. Livestock sales contributed 65% to on-farm income in type-4 farm households. Other farm enterprises viz*.* complementary and supplementary enterprises contributed 4% to on-farm income in type-2 farm households.

The off-farm income included wages for working as hired casual labourers in farms of wealthier neighbors, wages for doing unskilled manual work under Kerala Rural Employment Guarantee Scheme (KREGS), and wages for manual work under women’s labour collectives. KREGS operating under the Mahatma Gandhi National Rural Employment Guarantee Scheme (MGNREGS) of the Government of India, provides 100 days of guaranteed employment in a year to every adult household member in need of wage employment and desire to do manual or unskilled work in and around the village. Works related to building and maintenance of canals, renovation of ponds, wells, and farmland, afforestation, etc. are undertaken under KREGS. Many women in the study area, who are homemakers had come together to form women’s labour collectives, locally known as ‘Thozil Koottam’, to take up agricultural activities related to the cultivation of paddy, banana, tubers, coconut palm, and land terracing. Once these women exhaust the 100 days of work under MGNREGS, they move out to the open market as a collective to seek work in private lands in neighboring areas. For the landowners, this meant labour availability in the local market at a reasonable rate, at a time when it had become difficult to find labourers to work. In converse, in some areas during peak agriculture season, the farmers are experiencing shortage of labour due to government’s schemes like KREGS and MGNREGS leading to increased labour wages and cost of production. In addition, reduced participation of youths in agricultural activity also led to increased shortage of labour in agricultural activity^35^.

Non-farm income consisted of overseas remittances, running small businesses in the unorganized sector, and salary from the service sector. The proximity of the study area to the state capital provided educated youth in farm households with ample non-farm employment opportunities. Nevertheless, the dependence of farm households on off-farm and non-farm income was quite high since they contributed more than 65% to farm household income across all farm types (Table 1). Studies have shown that farm households are compelled to diversify their livelihood in times of shortfall in agricultural production^36,37^.

### Constraints to agricultural production identified for targeted farming systems interventions

The typology results had identified four farm types based on resource endowment and livelihood strategy (Table 1). The target group is the households in a farm type who rely on research findings for ideas and strategies to improve the way they do agriculture. For solving agricultural production problems, identification of constraints that work as a bottleneck by hindering the problem-solving process is a vital step, so that targeted farming systems interventions based on research findings can be made, enabling the farm household to push against that constraint and overcome it. Research-for-development programs seeking to sustainably intensify agricultural production in the target communities should take into account the opportunities and constraints identified across the farm types and tailor their development strategies, interventions and policies accordingly ^11^. Cost-effective socially acceptable farming systems interventions were envisaged based on production constraints identified in farm households in each farm type, to optimize resource utilization in households within a farm type, and also to promote resource flow and interactions between farm types, to ensure the stability of existing farming systems (Table 2). Farm typologies are classifications based on a set of criteria, and farm types are generally uniform in terms of these criteria, with some intra-group variation. As a result, typologies are useful for bringing together farmers for discussion so that groups of farmers who manage their farms similarly, have similar basic goals, or have similar constraints and possibilities can be formed^20,38^. The following sections reflect on production constraints identified and targeted farming systems interventions envisaged in each farm type.

**Table 2:** Constraints to agricultural production in farm types and farming systems interventions envisaged.

Problem encountered	Constraint identified	Constraint severity index (CSI) and rating					Farming systems interventions envisaged
		Farm type				Mean	
		1	2	3	4		
**Cropping system**							
*Rice*							
Crop loss	Pests: stem borer, rice bug, rodents	0.1 Very low	0.1 Very low	2.5 Medium	3.8 High	1.6 Low	Bird perches for increased activity of insectivorous and predatory birds; rational use of plant protection chemicals
Low yield	Traditional variety	0.0 None	0.0 None	3.8 High	2.6 Medium	1.6 Low	Introduction of high yielding variety
Low yield	Soil acidity; imbalanced fertilization	0.0 None	0.0 None	3.1 High	2.4 Medium	1.4 Low	Liming and rational use of fertilizers
Crop loss	Diseases: blast, sheath blight, sheath rot	0.1 Very low	0.1 Very low	2.7 Medium	1.4 Low	1.1 Low	Pseudomonas fluorescens for blast, sheath blight and sheath rot
Crop loss	Weeds	0.2 Very low	0.2 Very low	2.8 Medium	1.3 Low	1.1 Low	Stale seed bed for weed management
Low income from rice-rice-fallow cropping system	Limited water resources available for raising summer rice crop	0.0 None	0.0 None	1.7 Low	0.8 Very low	0.6 Very low	Liming and Rhizobium inoculated cowpea seeds in summer rice fallows
*Banana*							
Crop loss	Pest: banana rhizome weevil	4.2 Very high	3.7 High	2.8 Medium	2.6 Medium	3.3 High	Planting healthy sucker, removal of outer layer of rhizome and sun drying after smearing with cow dung slurry and ash
Excessive fertilisation	Soil acidity; imbalanced fertilization	3.9 High	3.3 High	2.6 Medium	2.5 Medium	3.1 Medium	Liming and rational use of fertilizers; incorporating green manure cowpea with 75 percent recommended dose of fertilizer for banana
Poor soil moisture conservation in summer	Lack of awareness of existing options	1.6 Low	2.9 Medium	1.5 Low	1.1 Low	1.8 Low	Mulching banana basin with banana residue for soil moisture conservation
*Cowpea*							
Crop loss	Disease: dry root rot	4.1 Very high	2.9 Medium	1.8 Low	1.4 Low	2.6 Medium	Seed treatment with Pseudomonas fluorescens; drenching and spraying with carbendazim
Low yield	Traditional variety	2.8 Medium	4.0 High	1.7 Low	1.2 Low	2.4 Medium	Introduction of high yielding variety
Excessive fertilisation	Soil acidity; imbalanced fertilization	1.3 Low	3.2 High	0.7 Very low	0.6 Very low	1.5 Low	Liming and Rhizobium inoculated cowpea seeds
*Cassava*							
Low yield	Traditional variety	3.8 High	3.5 High	2.2 Medium	2.8 Medium	3.1 High	Introduction of high yielding variety
Low yield	Imbalanced fertilization	2.9 Medium	4.1 Very high	1.8 Low	2.0 Low	2.7 Medium	Rational use of fertilizers
Low income from cassava cropping system	Lack of awareness of existing options	1.8 Low	2.4 Medium	0.5 Very low	0.2 Very low	1.2 Low	Intercropping cassava with cowpea; Liming and Rhizobium inoculated cowpea seeds
*Elephant Foot Yam*							
Low yield	Traditional variety	3.8 High	4.6 Very high	3.0 Medium	2.8 Medium	3.6 High	Introduction of high yielding variety
Low yield	Imbalanced fertilisation	2.7 Medium	3.4 High	1.6 Low	1.9 Low	2.4 Medium	Rational use of fertilizers
*Turmeric*							
Low yield	Traditional variety	2.7 Medium	1.5 Low	1.7 Low	1.9 Low	2.0 Low	Introduction of high yielding variety
Low yield	Imbalanced fertilisation	2.1 Medium	1.4 Low	1.6 Low	1.7 Low	1.7 Low	Rational use of fertilizers
*Coconut*							
Crop loss	Pest: rhinoceros beetle	2.6 Medium	2.6 Medium	3.7 High	3.4 High	3.1 High	Metarrhizium anisopliae application to breeding site; neem cake and sand application to leaf axil; naphthalene balls and sand application to leaf axil
Low yield	Soil acidity; imbalanced fertilization	2.7 Medium	3.3 High	2.1 Medium	2.2 Medium	2.6 Medium	Liming and rational use of fertilizers
Coconut palm residues like coconut leaves, crown waste, dried spathes, husk etc. are burnt in field	Lack of awareness of existing options	2.0 Low	2.7 Medium	2.3 Medium	2.4 Medium	2.4 Medium	Recycling of coconut palm residues by depositing them in small trenches 0.3 to 0.5 m deep at a distance of 2 to 2.5 m away from base of trunk
Low income from inter/mixed crops in coconut based multiple cropping system	Unutilized vacant interspaces	2.5 Medium	1.7 Low	2.4 Medium	2.5 Medium	2.3 Medium	Inter/mixed cropping with legume: cowpea; tuber: cassava, elephant foot yam; spice: turmeric; fruit: banana, papaya; fodder: bajra napier hybrid
Poor soil moisture conservation in summer	Lack of awareness of existing options	1.3 Low	1.7 Low	2.6 Medium	2.2 Medium	2.0 Low	Mulching coconut basins with coconut leaves at onset of northeast monsoon to add organic manure and to reduce soil temperature during summer
*Fodder*							
Low availability of green fodder	Traditional fodder variety	0.1 Very low	2.8 Medium	2.7 Medium	1.8 Low	1.9 Low	Introduction of high yielding fodder variety
*Other problems in cropping system*							
Vegetables for household purchased from local market	Lack of awareness of existing options	2.8 Medium	1.4 Low	3.1 High	3.2 High	2.6 Medium	Establishment of nutritional kitchen garden with brinjal /bhindi–cabbage/cauliflower /cowpea–amaranth /snakegourd /bittergourd crop sequence in growbags
Fruits for household purchased from local market	Lack of awareness of existing options	1.9 Low	1.6 Low	2.5 Medium	2.7 Medium	2.2 Medium	Introduction of high yielding papaya in vacant spaces in backyard
Crop residues burnt in field for clean cultivation	Lack of awareness of existing options	1.4 Low	2.5 Medium	2.7 Medium	2.2 Medium	2.2 Medium	Earthworms for vermicomposting crop residues that are usually burnt for clean cultivation
Overgrown perennial trees	Lack of awareness of existing options	2.3 Medium	0.2 Very low	1.5 Low	1.6 Low	1.4 Low	Shade regulation through lopping of branches of perennial trees
**Livestock system**							
*Dairy cattle*							
Mastitis resulting in low milk yield and inflammation of udder	Lack of awareness of existing options	0.1 Very low	3.8 High	3.2 High	2.6 Medium	2.4 Medium	Disinfection of milkers’ hands, udder washing with sanitizing solution, post milking teat sanitation
Low fat content of milk	Lack of awareness of existing options	0.1 Very low	2.4 Medium	2.6 Medium	1.9 Low	1.8 Low	Inclusion of mineral mixture in feeding schedule
*Layer chicken*							
Low egg production	Non-descript desi chicken breeds	1.9 Low	2.5 Medium	0.6 Very low	0.7 Very low	1.4 Low	Introduction of improved high egg laying birds
*Other problems in livestock system*							
High cost of feed concentrate	Lack of awareness of existing options	4.2 Very high	3.6 High	2.3 Medium	2.6 Medium	3.2 High	Establishment of azolla plot and inclusion of azolla in feeding schedule
Malnourishment and impaired health	Lack of awareness of existing options	3.0 Medium	2.1 Medium	2.8 Medium	2.3 Medium	2.6 Medium	Regular deworming
**Agro-processing and value addition**							
Low price for harvested coconuts	Lack of value addition	2.8 Medium	3.0 Medium	2.3 Medium	2.4 Medium	2.6 Medium	Dehusking, grading and local marketing of coconut
Milk sold to dairy cooperative fetches low price	Lack of value addition	0.2 Very low	1.1 Low	1.4 Low	2.9 Medium	1.4 Low	Local direct marketing of milk
Low price for harvested paddy	Lack of value addition	0.1 Very low	0.1 Very low	3.2 High	2.3 Medium	1.2 Low	Milling and local marketing of rice

*Farm household:* Farm household is the centrepiece of the farming system. Improvements in the existing farming system involve intensification, diversification, and an increase in the operational area of the farm household. Crop-livestock farming systems are the backbone of small-holder agriculture in developing countries^39^. The largest share of surveyed farm households comprised of resource-constrained type-1 households 46.5% having limited access to land (Table 1). The rest of the households though had marginally higher land availability offers little scope for increasing agricultural production through land area expansion. Kerala with a high literacy rate of 94% has the highest overall life expectancy at birth, at 72 years for men and 78 years for women ^40^ (GoK, 2019). Household heads in all surveyed households were elderly males aged 60 years who are the decision-makers in the utilization of household land for agricultural activities (Table 1). Targeted farming systems interventions envisaged for intensification and diversification of existing farming system, therefore must be pragmatic and problem-solving to find acceptance among the increasingly aging household head, who tend to show reluctance towards drastic changes in the existing farming system.

Dependence on off-farm and non-farm income was quite high among all surveyed households (Table 1). Only one out of four household members in each surveyed household were found working on-farm. Scarcity of household labour and the high cost of hired labour is likely to hamper efforts at diversification into supplementary enterprises having low-profit margins like a nutritional kitchen garden, except as part of increased awareness of health benefits to household members. Similarly, households are less likely to intensify existing rice-rice-fallow cropping system with legume cowpea in summer fallow and stop burning of crop residues in the field for clean cultivation, except as part of increased awareness about soil health and environmental pollution respectively (Table 2). Targeted farming systems interventions were therefore envisaged to be delivered through a capacity building and training program, to bring about a change in knowledge, attitude, and skill of the farm household for efficient farm operations.

*Foodgrain:* Rice was the major foodgrain in the study area. Constraints of high severity in a type-3 household that had the largest area under food grain were low yield due to traditional variety, soil acidity, and imbalanced fertilization (Table 2). Crop loss due to pests was a constraint of high severity in type-4 households. The stale seedbed for weed management was the farming systems intervention envisaged to manage weeds in rice, which was a constraint of medium severity in the type-3 household. Farming systems intervention envisaged in summer rice fallow was raising cowpea utilizing the limited water available during the season. In general, the agricultural activity of Kerala is affected by limited water availability during winter *rabi* and summer season, poor soil fertility due to low nutrient holding capacity of the soil, inadequate crop protection, non-availability of quality seed material, and increased cost of cultivation. The farmers need to adapt soil test based fertilizer recommendation to meet the crop nutrient demand for reducing yield gap. Suitable pest and weed management are very much necessary to combat the crop loss. Adaption of climate resilient improved cultivars, bringing more area under irrigation, intercropping, crop rotation, and mulching are imperative to increase food grain production and to achieve food security of small and marginal farmers^41^.

*Horticulture:* Banana, cowpea, cassava, and elephant foot yam were the widely cultivated fruit and vegetable in the study area (Table 2). Crop loss due to pests in banana and disease in cowpea were constraints of very high severity in type-1 households. The constraint in fruit and vegetable production due to traditional variety and imbalanced fertilization were of high to very high severity in type-2 households, which had a large area under fruit and vegetable. Raising cowpea is envisaged in farming systems interventions to utilize vacant interspaces of cassava and thus substantially lower the nitrogen fertilizer requirement of cassava. Cultivation of traditional poor-yielding turmeric varieties along with imbalanced fertilization were constraints of medium severity in the type-1 household (Table 2). Coconut was an important plantation crop in the study area, occupying the substantial cropped area in type-2 households (Table 2). Soil acidity and imbalanced fertilization were constraints of high severity in coconut in type-2 households. Crop loss in coconut due to pests was a constraint of high severity in type-3 and 4 households. Low green fodder availability due to poor yielding traditional fodder variety was a constraint of medium severity in type-2 and 3 households (Table 2). A multi-storeyed cropping system having cowpea, cassava, elephant foot yam, turmeric, banana, papaya, and fodder was the farming systems intervention envisaged to effectively utilize vacant interspaces of coconut. The Kerala state is major spice cultivating state and majority of the small, medium and large farmers are actively involved in the spice and plantation crops cultivation. The high value of spice and plantation crops is attracting rural youths also into horticulture sector, especially in processing of spices and their export to Gulf and European market. Kerala government is also promoting organic spice production to boost the local and international organic market for their products. In addition, Kerala's home gardens are typical examples of low to medium-input sustainable agroecosystems. Home gardens are assemblages of plants, which may include trees, shrubs, and herbaceous plants that grow in or close to a homestead, are planted and managed by members of the household, and the products and services are primarily for household consumption. These home gardens are having great importance in meeting farm family food and nutritional security^35^.

*Livestock:* Low milk yield in dairy cattle due to lack of awareness about mastitis infection was a constraint of high severity in type-2 and 3 households (Table 2). Raising awareness about hygiene to prevent mastitis and inclusion of mineral mixture in feeding schedule to increase milk fat content are the farming systems interventions envisaged for dairy cattle. Poor egg production in layer chicken due to rearing of non-descript desi chicken breed was a constraint of medium severity in the type-2 household (Table 2). Regular deworming was the farming systems intervention envisaged to improve livestock health in all households (Table 2). The dairy farmers of Kerala are experiencing several problems like high cost of veterinary service and medicine, high cost of cattle feed ,non-availability of green and dry fodder round the year, high labour cost, lack of need based training, non-availability of high yielding milch animals^42^. The government and Veterinary department of Kerala needs to address these issues to boost the livestock production and farmers income.

*Complementary enterprises:* Complementary enterprises in a system support one another^43^. Vermicomposting and Azolla cultivation were the complementary enterprises envisaged in farming systems interventions. Crop residues interfering with field operations was a problem, with the farmer often resorting to burning crop residue in situ*,* causing loss of nutrients and organic matter to the soil. Lack of awareness about environmentally safe ways to manage crop residues was a constraint of low to medium severity in all households (Table 2). Promoting the use of crop residues for vermicomposting and as mulch in banana and coconut for soil moisture conservation were the farming systems interventions envisaged to discourage the burning of crop residues (Table 2). The establishment of the Azolla plot and inclusion of Azolla in the feeding schedule of livestock were envisaged in farming systems interventions to reduce feed cost (Table 2).

*Supplementary enterprises:* Supplementary enterprises in a system utilize the otherwise unutilized resources^43^. Nutritional kitchen garden, agro-processing, and value addition were the supplementary enterprises envisaged in farming systems interventions. Fruits and vegetables for household consumption were found purchased from the local market due to production shortfall within the household, which was a constraint of low to high severity in all households (Table 2). The establishment of the nutritional kitchen garden and the growing of fruit trees in the backyard were the farming systems interventions envisaged ensuring nutritional security to the household. Encouraging farmers to take control of agro-processing and local marketing of primary production to capture the value that is added to it, thus fetching a better price for the produce, was the farming systems intervention envisaged for coconut, paddy, and milk, as per their recorded severity of constraints in respective farm types (Table 2).

### Importance of public distribution system (PDS) for food distribution

The Public Distribution System (PDS) was created as a way to manage scarcity and distribute food grains at low rates. PDS has evolved into a key component of the government's food economy management strategy. PDS is a supplemental program that is not meant to meet a household's or a part of society's complete need for any of the commodities given under it. Historically, Kerala's agricultural production has been directed toward cash crops, rather than food crops such as rice and wheat. As a result, the problem of food scarcity in Kerala has worsened. PDS is becoming more important in Kerala, where population density is high and farming patterns are mostly dependent on rains, with no consistent irrigation infrastructure, causing food supply availability to fluctuate over time, resulting in uncertainty. In order to avoid such situations and maintain the supply of required commodities, a PDS system is essential. Kerala's below-poverty-line (BPL) households consume 40–55 percent of their rice through PDS. The PDS supplied a higher percentage of the rice requirements. It is also clear that rural areas have done marginally better than urban areas in terms of PDS system utilization. It is worth noting that in Kerala, about 80% of BPL households still have access to the PDS, even at various levels of utilization, thereby reducing the pressure on local farmland^44^.

## Conclusion

Based on the results, it can be concluded that farming system typology identified four distinct farm types in marginal households at southern coastal plains of Thiruvananthapuram district, Kerala state, India. The coexistence of diverse farm households within relatively homogenous farm types was characterized through farming system typology. Constraint analysis assessed the severity of constraints in food grain, horticulture, livestock, complementary and supplementary enterprises in these farm types, which allowed for targeted farming systems interventions to be envisaged to overcome constraints. Interventions planned through the identification of farm types strategy offered a glimpse of hope for boosting net return, lowering production costs, and increasing farm income in a comprehensive perspective that could be scaled up to farm types for realizing the benefits, particularly by resource-poor farmers. From a systems perspective, intervention planning based on identified constraints for different components of farming systems, such as field crops, vegetable production, and allied components, combined with multilevel interventions on farmers' fields, could allow farmers to increase their net income by a significant margin. Farming system typology together with constraint analysis is therefore suggested as a practical framework capable of identifying type-specific farm households for targeted farming systems interventions.

## Data availability

The data presented in this study are available on request from the corresponding author.
